# Structure of Arabic Scale of Death Anxiety With Chinese College Students: A Bifactor Approach

**DOI:** 10.3389/fpsyg.2018.02511

**Published:** 2018-12-12

**Authors:** Qiuyun Li, Yan Cai, Qingrong Tan, Dongbo Tu

**Affiliations:** School of Psychology, Jiangxi Normal University, Nanchang, China

**Keywords:** death anxiety, Arabic scale of death anxiety, Chinese college students, factor analysis, bifactor model

## Abstract

The Arabic Scale of Death Anxiety (ASDA), as one of the most widely used measures of death anxiety (DA), has increasingly been applied in many studies. However, the structures derived from different studies are highly inconsistent. In this study, both traditional and novel (bifactor) modeling approaches were used, to investigate the most optimal structure of the ASDA in a sample of 984 Chinese college students. After a series of comparisons, the results showed that the bifactor model, with a dominant general DA factor and three distinct sub-dimensions, was the most optimal measurement structure, and measurement invariance of this bifactor model between sexes was also confirmed. Based on the implications of this bifactor model, the discussion was focused mainly on whether distinct dimensions should be interpreted or not. Some strengths and limitations of the study were also discussed at the end of the paper.

## Introduction

From ancient times to the present, death has remained one of the main concerns for humans. Death anxiety is the negative relationship associated with death, such as the angst, fear, and dread of death or death-related events (Qiu et al., 2015; Yang et al., 2016). It is not a sudden emotion but a continuous state throughout life. It is a highly personal issue, depending on different events and situations and the degree of anxiety related to death, may vary from one person to the other (Bengtson et al., 1977). Mild death anxiety does not affect people's lives, but once it becomes excessive, it will cause great harm to one's physical and mental health which may result in depressive disorders, obsessive–compulsive disorders, schizophrenia, post-traumatic stress disorder, manic-depression, and eating disorders etc (Khanna et al., 1988; Thorson and Powell, 2000; Arndt et al., 2005; Chung et al., 2005; Strachan et al., 2007). Considering the various disorders listed above, understanding the nature and risk factors of DA has important clinical implications.

To evaluate the degree of an individual's death anxiety, some questionnaires were developed, such as Templer's Death Anxiety Scale (T-DAS), the Spanish Death Anxiety Inventory (SDAI), the Arabic Scale of Death Anxiety (ASDA), the Collett Lester Fear of Death Scale (CLFOD), and the Revised Death Anxiety Scale etc (Templer, 1970; Thorson and Powell, 1992; Abdel-Khalek, 2004; David, 2004; Tomás-Sábado et al., 2005). The ASDA scale has become one of the most widely used scales, as it can measure death anxiety from a multidimensional profile (Abdel-Khalek, 2004). More importantly, it has been applied in different circles, such as health care professionals (Ayyad, 2013), college students (Aydogan et al., 2015), drug dependents (Daradkeh and Moselhy, 2011), healthy individuals (Abdel-Khalek, 2005), middle-aged individuals (Abdel-Khalek and Al-Kandari, 2007) etc., and the total-score, based on these samples, all showed good reliability and validity. It has therefore been translated into other languages, including Turkish (Aydogan et al., 2015), English (Abdel-Khalek et al., 2009), and Chinese (Qiu et al., 2016).

However, all of these studies were conducted under the assumption that the ASDA scale has good conceptual and psychometrical assessment validity. Although many studies have explored the structure of the ASDA, it has been previously mentioned that death is complex and may have different meanings for different people (Karasu, 1985). People with different experiences, cultures and social statuses, may have distinct attitudes toward death. Thus, the original structure of the ASDA might not be valid for Chinese people. Moreover, even in the same cultural environment, subtle differences may exist between diverse groups. To make the measurements more valid and to ensure comparability of measured properties across different groups of respondents, good psychometric invariance and clear structure are necessary.

## Factor Structure of the ASDA

The ASDA scale is composed of 20 items and the response options for each item ranges from 1 to 5, representing the degree of variance ranging from “no” to “very much.” While the total score of the scale ranges from 20 to 100, the higher the score is, the higher the degree of death anxiety is to investigate the structure of the ASDA, some studies were done (Abdel-Khalek et al., 2009; Dadfar and Bahrami, 2016; Dadfar et al., 2016; Qiu et al., 2016), however, the structures proposed by these studies have been highly inconsistent and were mainly focused on 3-, 4-, and 5-factor structures.

For a 3-factor structure, Abdel-Khalek (2004) conducted the principal components analysis in a sample of undergraduates in three Arabic countries (Kuwait, Egypt, and Syria) and the Kaiser criterion (eigenvalues greater than one; Kaiser, 1960) was used to determine the number of the factor. The 3-factor structure, which accounted for 61.0% of the common variance (labeled the structure as “A”), was determined in the Kuwaiti sample, and the three factors were labeled as: (a) fear of dead people and tombs, (b) fear of lethal disease, and (c) fear of postmortem events. Then, another 3-factor structure was revealed based on the Kaiser criterion in the sample of elders (Dadfar et al., 2016), which accounted for 67.88% of the total variance (labeled as “B”). The three factors were different from model A, and were labeled as (a) fear of lethal disease and death, (b) fear of lethal disease and postmortem events, and (c) death fear. Recently, a 3-factor structure (labeled as “C”) which accounted for 57.09% of the total variance was revealed, using the same method as above, based on a Chinese sample composed by 589 hospital staff members and 768 university students (Qiu et al., 2016). The correlations between these factors ranged from 0.572 to 0.664. These factors were consistent with model A and the total-score of the ASDA in this sample showed good reliability (α = 0.91, 95% CI [0.90, 0.92]).

For a 4-factor structure, there were two different structures based on the Egyptian and Syrian sample and they were also derived from the Abdel-Khalek study, Abdel-Khalek (2004) using the same method as the Kuwaiti sample. In the Egyptian sample, the 4-factor structure (labeled as “D”) accounted for 62.1% of the total variance and the four factors were labeled as (a) fear of dead people and tombs, (b) fear of postmortem events, (c) fear of lethal disease, and (d) death preoccupation. In the Syrian sample, the 4-factor structure (labeled as “E”) accounted for 55.2% of the total variance. In this structure, the first three factors had the same name as model D, while the last factor was labeled “death ^..^concern.”

Dadfar and Bahrami (2016) introduced a 5-factor structure based on the Farsi Version of the ASDA Scale. Based on a sample of middle-aged people, five components with eigenvalues greater than one were retained. However, it was found that two factors lacked sufficient item representation, which means the number of items loaded on these factors was < 3 (Velicer and Fava, 1998; Maccallum et al., 1999; Faraci et al., 2013; Park et al., 2016). This solution was therefore not taken into consideration in the current article.

The pattern of the factor loadings from the five studies mentioned above are depicted in Table 1. It can clearly be seen that the pattern of item-loading on the first factor “fear of dead people and tombs” in model A, were relatively stable across most solutions. The items loaded on the “fear of lethal disease” factor and the “fear of postmortem events” factor in model A, was largely similar to those in model C, while they mostly loaded onto a common “fear of lethal disease and postmortem events” factor in model B. The third “death fear” factor in model B, was composed of the items from the “fear of dead people and tombs” factor and the “fear of lethal disease” factor in model A, largely similar to the “death preoccupation” factor in model D. As for the second “fear of lethal disease” factor in model A, there was great consistency in the 3-factor solution, while in the 4-factor solution, this dimension was split into two separate factors.

**Table 1:** Pattern of item to factor loadings across five existing structures of the ASDA.

**Item**	**Model A**			**Model B**			**Model C**			**Model D**				**Model E**			
	**Fear of dead people and tombs**	**Fear of lethal disease**	**Fear of postmortem events**	**Fear of lethal disease and death**	**Fear of lethal disease and postmortem events**	**Death fear**	**Fear of dead people and tombs**	**Fear of lethal disease**	**Fear of postmortem events**	**Fear of dead people and tombs**	**Fear of lethal disease**	**Fear of postmortem events**	**Death preoccupation**	**Fear of dead people and tombs**	**Fear of lethal disease**	**Fear of postmortem events**	**Death concern**
2	^*^			^*^			^*^			^*^				^*^		
3	^*^					^*^	^*^			^*^				^*^		
8	^*^			^*^			^*^			^*^				^*^		
11	^*^			^*^			^*^			^*^				^*^		
12	^*^			^*^			^*^			^*^				^*^		
16	^*^			^*^			^*^			^*^				^*^		
17	^*^			^*^			^*^			^*^				^*^		
18	^*^			^*^					^*^	^*^			^*^				^*^
1		^*^				^*^		^*^					^*^		^*^	
4		^*^			^*^			^*^			^*^				^*^	
5		^*^			^*^			^*^			^*^				^*^	
6		^*^			^*^			^*^			^*^					
10		^*^			^*^			^*^			^*^						^*^
14		^*^				^*^			^*^				^*^		^*^	
19		^*^			^*^			^*^			^*^						^*^
20		^*^				^*^		^*^					^*^				^*^
7			^*^		^*^				^*^			^*^				^*^
9			^*^				^*^					^*^				^*^
13			^*^	^*^	^*^				^*^			^*^				^*^
15			^*^		^*^				^*^			^*^				^*^

* *, primary item to factor loading within each model*.

The inconsistency between these studies can partly be attributed to the diversity of participants, therefore, to test whether an instrument has the same factor structure across different groups, it is necessary to conduct a confirmatory factor analysis. While it is worth mentioning that most of these structures were suggested based solely on an exploratory factor analysis (EFA), only Qiu's study carried out a confirmatory factor analysis (CFA). Furthermore, determining the number of factors in these studies was based on the Kaiser's criterion, which is the most widespread method because of its theoretical basis and ease of use. However, some researchers have suggested that it is problematic to make a decision based on this method solely, as it typically overestimates the number of components (Cattell and Jaspers, 1967; Browne, 1968; Lee and Comrey, 1979; Zwick and Velicer, 1986).

Although there are many existing structures, lack of structural verification, and factor retention methods used, makes the validity and representativeness of these factors difficult to accept. Thus, the first goal of this study was to find the best representation structure of death anxiety as measured by the ASDA, on the sample of Chinese college students by systematically testing and comparing the fit of these existing structures.

## Bifactor Approach

The bifactor model was originally developed by Holzinger and Swineford (1937), and was looked as an alternative model to non-hierarchical multidimensional models. The details of the model are listed as follows:

If a scale is conducted with *p* items, the score of each item is presented as *x*_1_, *x*_2_, ⋯ , *x*_*p*_, and this scale has measured a general factor G and *n* specific factors *F*_1_, *F*_2_, ⋯ , *F*_*n*_, then the observed variable *x*_*i*_ can be presented as:

$$\begin{array}{r} x_{i}=a_{i}G+\overset{n}{\underset{j=1}{\sum }}b_{ij}F_{j}+\delta_{i},i=1,2,\cdots \;,p, \end{array}$$

where *a*_*i*_ is the loading of item *i* on the general factor G, *b*_*ij*_ is the loading of item *i* on the specific factor *F*_j_, δ_*i*_ is the measurement error of *x*_*i*_. Furthermore, to make the model easier to converge and explain, it is usually assumed that the relationship between the general factor, specific factors and measurement error, are orthogonal.

As shown above, it differs from the traditional structure, in that it could separate the specific factors from the general factor. That means, in a traditional model, the correlation between items are attributed to the influence of multiple correlated traits, while the bifactor model states the covariation of items because they share a common trait, while the independent part of the covariation is possibly due to shared item content (Reise et al., 2007). Thus, we can examine the contribution of an item from two aspects (i.e., the loadings on the general factor and the specific factor), after the common variance shared with other items has been taking into account by the general factor. Additionally, the percent of common variance due to the general factor, is also important to consider, in order to decide whether a multidimensional structure is suitable or not. Because the bifactor model provided richer and clearer information, it attracted increased attention from researchers (Brown et al., 2011; Golay and Lecerf, 2011; Tay et al., 2011; Gignac and Watkins, 2013). In this study, the bifactor model was also applied as an alternative structure of the ASDA, not only to solve the inconsistencies in previous studies, but also to provide researchers with a new perspective and more information to understand the underlying factor structure of the ASDA.

## Method

### Participants

Initially, 1,038 college students were recruited from three different colleges in three different Chinese provinces: Tianjin, located in northern China, with a total of 252 participants (43.3% male, 56.7% female); Henan, in central China, with a total of 324 participants (47.5% male, 52.5% female); and Jiangxi, in southeast China, with a total of 462 participant (53.7% male, 46.3% female). After introducing the purpose and content of the study and obtaining informed consent from the subjects, all participants completed the study materials in a class under the supervision of the investigators and received a small honorarium as an incentive for their participation. After strict screening, 28 participants were excluded because of substantial missing data (i.e., the proportion of missing data exceeds 50%), and 26 participants were excluded as their responses showed obvious casualness. Finally, a total of 984 usable questionnaires remained and the effective response rate was 94.8%. Among these respondents, 478 were males, and 506 were females, the range of age was 16–26 years (*M* = 20.65; SD = 1.465), and there were no significant sex differences on the age with *t* (982) = −0.084 and its *p* = 0.933. Additionally, 56.5% of these participants come from the countryside, 23.4% come from towns and 20.1% come from cities. As for the family status, 26.2% were the only-child while the remaining 73.8% were not.

The current research was carried out following the recommendations of psychometric studies on mental health, at the Research Center of Mental Health, Jiangxi Normal University, and was approved by the Research Center of Mental Health, Jiangxi Normal University, and the Ethics Committee of Jiangxi Normal University. The written informed consent was obtained from all participants in accordance with the Declaration of Helsinki.

### Measures

#### The Arabic Scale of Death Anxiety—Chinese Version [ASDA(C)]

The Arabic Scale of Death Anxiety (ASDA) was developed by Abdel-Khalek in 2004, and is one of the most commonly used instruments for assessing death anxiety or the fear of death and has also been developed into a Chinese version in 2016 (Qiu et al., 2016). In this study the Arabic Scale of Death Anxiety—Chinese Version was used, which includes 20 short statements with each item on a 5-point Likert-type format, ranging from 1 (no) to 5 (very much), with higher scores indicating higher death anxiety.

#### The Chinese Version of the Templer-Death Anxiety Scale [CT-DAS]

The Chinese version of the Templer-Death Anxiety Scale was conducted in this study as an external validation criterion. CT-DAS was translated from the English version T-DAS (Templer, 1970) and was developed into a Likert-type death anxiety scale (Yang et al., 2013). It contains 15 items and each item is rated on a 5-point Likert scale from 1 (completely disagree) to 5 (completely agree). The total score ranges from 15 to 75 and higher scores indicate higher levels of death anxiety. The reliability of the total-score in the study is acceptable (α = 0.709, 95% CI [0.68, 0.74]; Bland and Altman, 1997).

### The Procedure of Analyses

In order to investigate the structure of the ASDA (C), three sets of analyses were conducted in this study. All exploratory factor analysis was conducted using R (psych and GPArotation packages) and all confirmatory factor analysis was conducted using Mplus7.0 (Muthén and Muthén, 2012).

In the first set of analyses, a CFA analysis was conducted based on all data, to confirm six existing structures of the ASDA and to assess whether these existing structures would represent the structure of death anxiety best, as measured by the ASDA. Five of these structures are introduced in section Factor Structure of the ASDA and the other is the single-factor structure.

To take all possible structures into account, a second set of analysis was conducted. The sample was split randomly into two separate groups and one was subjected to the EFA while the other was subjected to the CFA analysis, to detect whether there were any new structures. Before the EFA, the KMO's Test of Sampling Adequacy and Bartlett's Test of Sphericity was used to assess whether factor analysis is feasible for the data. Generally, the values of the KMO's Test of Sampling Adequacy are >0.9 for excellent, between 0.50 and 0.60 for barely acceptable, and < 0.50 for unacceptable while a significant result of the Bartlett's Test of Sphericity indicates that the data is suitable for the EFA.

In EFAs, determining the number of factors is the most important issue and many factor retention methods have been developed. Kaiser's criterion and the Scree Test (Cattell, 1966) are the two most popular methods as they are easy to conduct using software such as SPSS and SAS. The parallel analysis (PA; Horn, 1965) can provide accurate results by comparing the eigenvalues derived from the actual data, to the eigenvalues derived from the random data, while factors are retained if the eigenvalue from the actual data exceeds the eigenvalues from a large number of random data (O'Connor, 2000). Although PA is one of the most accurate methods, it is risky to make a decision relying solely on this method, because of its slight tendency to recommend retaining too many factors (Hayton et al., 2004). Velicer's minimum average partial (MAP; Zwick and Velicer, 1986) could achieve about the same accuracy of the PA. Additionally, these methods can complement each other well with the PA, as the MAP is contrary to the PA and has a tendency to underestimate factors. The Comparison Data method (CD; Ruscio and Roche, 2012) is a variant of the PA, rather than generating random data sets, this technique creates and analyzes comparison data (CD) with a known factorial structure, to determine the number of factors to retain. It was proven to have superior performance over all other factor retention methods, including the PA, in previous studies. To make decision with caution, both the conventional methods (i.e., Kaiser's criterion and the Scree Test) and more robust empirical criteria (i.e., MAP, PA, and CD) were used in the present study.

Lastly, a series of bifactor CFA analyses were performed on all the structures mentioned above (due to the limitations of bifactor model, single-factor model was excluded) and the results of the bifactor CFA will be compared with the traditional model-fit. After a series of comparisons, the best structure representative was determined. Given adequate fit, the explained common variance (ECV) and percentage of uncontaminated correlations (PUC) were calculated to evaluate the degree of unidimensionality of the ASDA. The ECV was equal to the percent of common variance, due to the general factor in a bifactor model, the value of the ECV varied between 0 and 1. The greater the value is, the higher the degree of unidimensionality is. The percentage of uncontaminated correlations (PUC) is interpreted along with the ECV, which represents the percentage of the ASDA item correlations contaminated by variance, attributed to both the general and group factors.

Furthermore, both ω and ω-hierarchical were used to assess scale score reliability for these factors. ω estimate is an index for the proportion of variance, accounted for by a factor relative to the total observed score variance. It is more appropriate for tests of multidimensionality than for a coefficient alpha. In a bifactor model, each item response is assumed to be influenced by both the general factor and specific factors, the ω-hierarchical estimate could test the reliability of those factors, while controlling the variances accounted for in the other. The use of ω and ω-hierarchical in conjunction, could provide more information for the current study, as large discrepancies between ω and ω-hierarchical for subscales indicate that the interpretation of the subscales is not informative, due to the general factor.

### The Goodness-of-Fit Indexes

In this paper, four common goodness-of-fit indexes were used to evaluate the fitness of the factor structures, to data. They are the ratio of chi-square to degrees of freedom (χ^2^/df), Comparative Fit Index (CFI), the Tucker-Lewis index (TLI), and the Root Mean Square Error of Approximation (RMSEA) with its 90% confidence interval (90% CI). The criteria for these indicators are as follows: χ^2^/df < 5, CFI ≥0.90, TLI ≥0.90, and RMSEA ≤ 0.08 for an acceptable fit; χ^2^/df < 3, CFI ≥0.95, TLI ≥0.95, and RMSEA ≤ 0.05 for a good fit.

Additionally, the chi-square difference test was also used to compare between structures. The lower the value of Chi-square is, the better the fitness of the structure is. It is necessary to note that the Chi-square test is sensitive to the size of the sample, and it is may be significant when the actual difference between the observed model and the implied model covariance is small.

## Result

### Descriptive Characteristics

Descriptive statistics of the ASDA items in the sample of 984 college students are presented in the Table 2. The mean score of the ASDA was 51.21(SD = 14.50), 47.79 (SD = 13.47) for males and 54.44(SD = 14.71) for females. In addition to item 13 and item 14, there were significant sex differences on the items. Except for the table shown below, the correlations between the items were also tested. Almost all correlations were significant at p < 0.05, except for the associations between item 6, item 13 and item 14. The correlations between these items ranged from 0.07 to 0.81, and the average absolute association was 0.35, while the item-scale correlations ranged from 0.37 to 0.75 (the lowest item-total correlation for item 6 with *r* < 0.40).

**Table 2:** Descriptive statistics for the ASDA items in a sample of 984 Chinese college students.

**Item**	**M(SD) Total**	**M(SD) Male**	**M(SD) Female**	**Skewness**	**Kurtosis**	***T***	**Cohen'd**
Y1	2.14 (0.95)	2.01(0.95)	2.25(0.95)	0.96	0.72	−3.98^**^	0.25
Y2	2.81 (1.21)	2.48(1.08)	3.12(1.24)	0.47	−0.90	−8.76^**^	0.56
Y3	2.55 (1.19)	2.25(1.05)	2.83(1.24)	0.60	−0.57	−7.95^**^	0.51
Y4	2.69 (1.10)	2.43(1.01)	2.94(1.12)	0.52	−0.57	−7.57^**^	0.48
Y5	2.59 (1.21)	2.46(1.20)	2.72(1.22)	0.55	−0.69	−3.42^**^	0.22
Y6	3.95 (1.08)	3.82(1.09)	4.08(1.05)	−0.61	−0.94	−3.80^**^	0.24
Y7	2.28 (1.22)	2.14(1.16)	2.42(1.25)	0.76	−0.37	−3.71^**^	0.24
Y8	2.85 (1.21)	2.50(1.08)	3.17(1.24)	0.43	−0.94	−9.05^**^	0.58
Y9	2.46 (1.27)	2.23(1.16)	2.68(1.32)	0.58	−0.72	−5.70^**^	0.36
Y10	3.23 (1.21)	3.03(1.20)	3.42(1.18)	0.03	−1.20	−5.15^**^	0.33
Y11	2.39 (1.18)	2.16(1.11)	2.59(1.20)	0.72	−0.34	−5.85^**^	0.37
Y12	2.46 (1.13)	2.25(1.04)	2.65(1.18)	0.71	−0.27	−5.59^**^	0.36
Y13	2.05 (1.11)	2.06(1.10)	2.03(1.12)	0.89	−0.05	0.47	–
Y14	1.74 (1.02)	1.71(0.98)	1.78(1.06)	1.40	1.28	−1.13	–
Y15	2.26 (1.15)	2.18(1.09)	2.35(1.19)	0.78	−0.17	−2.37^*^	0.15
Y16	2.23 (1.07)	2.13(1.05)	2.32(1.09)	0.84	0.10	−2.76^*^	0.18
Y17	2.39 (1.04)	2.24(1.00)	2.53(1.06)	0.85	0.17	−4.40^**^	0.28
Y18	2.15 (1.01)	2.01(0.96)	2.27(1.04)	0.97	0.60	−4.14^**^	0.26
Y19	3.11 (1.22)	2.97(1.24)	3.25(1.18)	0.10	−1.12	−3.63^**^	0.23
Y20	2.88 (1.23)	2.73(1.22)	3.02(1.23)	0.40	−0.95	−3.74^**^	0.24
Total	51.21(14.50)	47.79(13.47)	54.44(14.71)			

T, Student's t-test with sex as the independent variable and item score as the dependent variable.

* P < 0.05;

** *P < 0.01*.

### Testing Existing Structures

Because the items were categorical, and the Mardia's (1970) test of multivariate kurtosis was indicative of nonnormality (z = 50, *p* < 0.001), the mean and variance-adjusted weighted least squares estimation, was performed as the CFA analysis method to test the fitness of five existing structures and a single-factor structure, with data from the full sample (*N* = 984). The results are presented in Table 3. The relative terms indicated that the structure C revealed by Qiu et al. (2016) fitted the data best (χ${}_{\left(167\right)}^{2}$ = 2900.492^*^). And the single-factor model fitted the data least (χ${}_{170}^{2}$ = 4267.572^*^). However, as for the absolute terms (i.e., based on the value of χ^2^/df, RMSEA, CFI and TLI), none of these models provided an acceptable fit to the data in this sample.

**Table 3:** CFA model-fit results for the existing structures of the ASDA(C) (*N* = 984).

**Model**	**No. of factors**	**χ^**2**^**	**df**	**χ^**2**^/df**	**RMSEA(90 %CI)**	**CFI**	**TLI**
Single-factor	1	4267.572^**^	170	25.10	0.157(0.152, 0.161)	0.827	0.807
A (Abdel-Khalek, 2004)	3	3399.213^**^	167	20.35	0.140(0.136, 0.144)	0.864	0.845
B (Dadfar and Bahrami, 2016)	3	3774.014^**^	148	25.50	0.158(0.153, 0.162)	0.838	0.813
C (Qiu et al., 2016)	3	2900.492^**^	167	17.37	0.129(0.125, 0.133)	0.885	0.869
D (Abdel-Khalek, 2004)	4	3317.154^**^	164	20.23	0.140(0.136, 0.144)	0.867	0.846
E (Abdel-Khalek, 2004)	4	3553.327^**^	165	21.54	0.144(0.140, 0.149)	0.861	0.840

* P < 0.05;

** *P < 0.01*.

### Alternative Structures

From the CFA analysis, it was assumed that a new multidimensional structure might exist. Thus, the data was divided into two halves randomly and subjected to the EFA and CFA analysis, respectively. The KMO's Test of Sampling Adequacy was 0.929 and the Bartlett's Test of Sphericity ($\chi_{190}^{2}$ = 10172.98) was significant (*P* < 0.001), indicating that the ASDA was appropriate for a factor analysis.

In the present study, we selected principal axis factoring with oblique rotation as the method of factor extraction and the cut-off of the factor loadings was 0.40 (i.e., 16% of the common variance). Five factor retention methods, mentioned before, were conducted to determine the number of factors. According to the result from the PA, five factors were retained and the results from the factor rotation are presented in the left panel in Table 4. While the number of factors from the PA was obviously over-defined as three items failed to load 0.40 or greater on any factor and two factors lacked sufficient item representation (one factor had no item significantly loaded on and one factor comprised of only two indicators).

**Table 4:** Item-factor loadings from EFA for the five-factor solution and three-factor solution.

**Items**		**5-factor solution**						**3-factor solution**	
	**Factor 1**	**Factor 2**	**Factor 3**	**Factor 4**	**Factor 5**	***h^**2**^***	**Factor 1**	**Factor 2**	**Factor 3**	***h^**2**^***
Y1	0.12	**0.57**	0.06	−0.02	−0.20	0.39	−0.02	**0.51**	0.12	0.33
Y2	**0.95**	−0.15	−0.07	0.09	−0.03	0.76	**0.97**	−0.18	−0.02	0.73
Y3	**0.92**	−0.05	−0.09	−0.06	0.04	0.68	**0.93**	−0.11	−0.08	0.65
Y4	**0.58**	0.04	0.14	0.01	−0.09	0.45	**0.52**	0.00	0.19	0.42
Y5	0.16	0.28	**0.40**	−0.05	−0.09	0.42	0.06	0.13	**0.43**	0.39
Y6	0.02	−0.22	**0.59**	−0.01	0.10	0.29	0.06	−0.23	**0.57**	0.27
Y7	0.03	**0.46**	0.14	−0.02	0.23	0.44	0.15	**0.48**	0.08	0.41
Y8	**0.92**	−0.16	0.01	−0.04	0.11	0.74	**0.99**	−0.21	−0.01	0.73
Y9	0.37	0.27	0.09	−0.11	0.31	0.54	**0.51**	0.20	0.00	0.49
Y10	0.08	−0.03	**0.68**	−0.07	0.17	0.52	0.14	−0.06	**0.63**	0.48
Y11	0.35	0.16	−0.04	0.19	0.34	0.65	**0.63**	0.26	−0.09	0.60
Y12	**0.50**	0.15	−0.04	0.11	0.24	0.63	**0.69**	0.21	−0.08	0.63
Y13	−0.13	**0.82**	−0.06	−0.08	0.09	0.51	−0.13	**0.83**	−0.10	0.50
Y14	−0.06	**0.96**	−0.20	−0.07	−0.04	0.64	−0.14	**0.94**	−0.20	0.61
Y15	−0.05	**0.56**	0.10	0.08	0.08	0.45	0.01	**0.62**	0.09	0.46
Y16	0.17	0.28	−0.13	0.32	0.19	0.51	**0.41**	**0.43**	−0.12	0.48
Y17	0.05	−0.21	0.14	**0.78**	0.06	0.63	0.38	0.09	0.25	0.40
Y18	0.01	0.24	0.02	**0.64**	−0.09	0.65	0.20	**0.45**	0.12	0.50
Y19	−0.15	0.00	**0.86**	0.14	−0.10	0.70	−0.20	0.02	**0.95**	0.71
Y20	−0.11	0.17	**0.69**	0.10	−0.07	0.58	−0.16	0.18	**0.75**	0.59

*h^2^, communality. The bold values indicate salient factor loadings*.

As a consequence of these findings, the results of other methods may provide a valuable reference. According to the MAP, three factors were retained when the minimum average partial was reached. In the CD, although the largest number of factors was set to 5, the results still indicated that the number of factors that should be retained, is 3. The Kaiser's criterion and the Scree Test supported this result. This 3-factor solution could account for 58.54% of the total variance and the eigenvalues of the three factors were 8.22, 1.87, and 1.62. The pattern of factor loadings for this solution, from oblique rotation, is presented in the right panel of the Table 4. As Table 4 shows, except for item 16 (“I get upset by witnessing a funeral”), which presented cross-loadings (the loadings on the primary factor and on at least one of the other factors exceeded.4) and item 17 (“The sight of a dying person frightens me”), which did not produce loadings higher than.4 on any factors, were eliminated, the remaining 18 items loaded moderately to strongly (0.43–0.97) on one factor. Each factor was well defined by 5–7 items and the communalities of these items ranged from 0.27 to 0.73. Thus, we finally determined a 3-factor solution (labeled the structure as “F”) for ASDA.

Although there were some subtle differences in the primary items to factor loading, the extracted factor structure was similar to that of previous studies. According to the factor names of the existing structures, these three factors were named “fear of dead people and tombs,” “fear of postmortem events” and “fear of lethal disease.”

To validate the fitness of the newly derived structures from the EFA, the CFA analysis was then conducted based on the remaining data. The results showed acceptable goodness-of-fit indexes for this three-factor solution according to the CFI and TLI (χ${}_{\left(132\right)}^{2}$ = 935.273^*^; *P* < 0.001, CFI = 0.919, TLI = 0.907, RMSEA = 0.118 [90% CI.113–0.123], and χ^2^/df = 7.09).

### Bifactor CFA

After the traditional CFA, we left the four latent variables uncorrelated, adhering to the principles of the bifactor model, to detect if a bifactor structure would provide a better account of the observed relationships than the traditional structure. A series of bifactor CFAs was performed to test the fit of not only the newly derived structure but also all structures (see Table 5). The result revealed that the bifactor model, based on model F (χ${}_{\left(117\right)}^{2}$ = 707.108^**^; *p* < 0.001, CFI = 0.973, TLI = 0.965, RMSEA = 0.072 [90% CI. 067–0.077], and χ^2^/df = 6.04), demonstrated a good fit according to the CFI and TLI, and an acceptable fit according to RMSEA. Although the χ^2^/df was slightly high, it was acceptable due to the sensitivity of the chi-square statistic to the sample size and the large sample used in the current study. Within the corresponding bifactor model of existing structures, model C from the 2016 study by Qiu et al. (χ${}_{\left(150\right)}^{2}$ = 1331.662^**^; *p* < 0.001, CFI = 0.950, TLI = 0.937, RMSEA = 0.089 [90% CI.085–0.094], and χ^2^/df = 8.88), demonstrated the best model fit with the lowest χ^2^ value and provided a good fit to the data, according to the CFI and an acceptable fit according to the TLI, but not according to the χ^2^/df and RMSEA.

**Table 5:** Bifactor CFA model-fit results for all the structures of the ASDA (*N* = 984).

**Model**	**χ^**2**^**	**df**	**χ^**2**^/df**	**RMSEA(90 %CI)**	**CFI**	**TLI**
A (Abdel-Khalek, 2004)	1794.138^**^	150	11.96	0.106 (0.101, 0.110)	0.931	0.912
B (Dadfar et al., 2016)	3247.866^**^	152	21.37	0.144(0.140, 0.148)	0.869	0.837
C (Qiu et al., 2016)	1331.662^**^	150	8.88	0.089(0.085, 0.094)	0.950	0.937
D (Abdel-Khalek, 2004)	2655.180^**^	150	17.70	0.130 (0.126, 0.135)	0.894	0.866
E (Abdel-Khalek, 2004)	2090.196^**^	152	13.75	0.114(0.110, 0.118)	0.918	0.898
F (This article)	707.108^**^	117	6.04	0.072(0.067, 0.077)	0.973	0.965

* P < 0.05;

** *P < 0.01*.

After model comparison, the bifactor model based on model F was the best account of the ASDA, after comparing the chi-square difference with the correlated-traits model F (Δχ^2^ = 228.165^*^, Δdf = 15) and the bifactor model based on model C (Δχ^2^ = 624.554^*^, Δdf = 33). The item-factor loadings from the standardized bifactor CFA solution of the best fit model, is presented in Table 6. The factor loadings revealed that each item loaded moderately to strongly (0.48 −0.76) on the general factor except for item 6 (0.28) and each group factor was defined by 2–4 items. All CFA loading estimates were statistically significant (*p* < 0.05). Within the bifactor model, the PUC was 70% and the ECV of the general factor was 63% and were 14, 9, and 15% for three specific factors. The reliability of the summed total scale score was 0.80, which suggested that 80% of the variance of the summed total scale score was attributable to the general factor. After controlling for the general factor, the reliabilities for the summed subscale scores were 0.22, 0.25, and 0.43. That meant that only a small part of the variance in the subscale scores could be explained by the specific factors, beyond what was already accounted for by the general factor.

**Table 6:** Bifactor structure of the ASDA: Item-factor loadings from standardized CFA solution (*N* = 984).

**Item number**	**Bifactor CFA^a^**			
	**General factor**	**Group factor1**	**Group factor2**	**Group factor3**
Y2	0.61(0.03)	0.70(0.02)	
Y3	0.64(0.02)	0.54(0.03)	
Y4	0.63(0.02)	0.25(0.03)	
Y8	0.62(0.03)	0.69(0.02)	
Y9	0.74(0.02)	0.18(0.04)	
Y11	0.76(0.02)	0.25(0.03)	
Y12	0.76(0.02)	0.34(0.03)	
Y1	0.55(0.03)		0.31(0.04)
Y7	0.66(0.02)		0.25(0.03)
Y13	0.48(0.03)		0.62(0.03)
Y14	0.52(0.03)		0.70(0.03)
Y15	0.60(0.02)		0.37(0.03)
Y18	0.74(0.02)		0.18(0.04)
Y5	0.62(0.02)			0.24(0.03)
Y6	0.28(0.03)			0.46(0.04)
Y10	0.56(0.02)			0.44(0.03)
Y19	0.50(0.03)			0.79(0.03)
Y20	0.58(0.02)			0.55(0.03)
ω	0.94	0.91	0.83	0.80
*ω_*h*_*	0.80	0.22	0.25	0.43
ECV	0.63	0.14	0.09	0.15

*group factor1, “fear of dead people and tombs”; group factor2, “fear of postmortem events”; group factor3, “fear of lethal disease”*.

a *All CFA loading estimates were significant at p < 0.05*.

Finally, the data was divided into male and female groups and then subjected to the bifactor CFA, respectively. The results showed that the fit of this model was good both in the male (χ${}_{\left(117\right)}^{2}$ = 389.439^*^; *p* < 0.001, CFI = 0.974, TLI = 0.966, RMSEA = 0.070 [90% CI.062–0.078] and χ^2^/df = 3.33) and female group (χ${}_{\left(117\right)}^{2}$ = 416.834^*^; *p* < 0.001, CFI = 0.972, TLI = 0.963, RMSEA = 0.071 [90% CI.064–0.079] and χ^2^/df = 3.56). At the same time, the fit indices that supported this factor structure was invariant between the male and female group, because all CFI differences were < 0.01 (ΔCFI = 0.002, ΔTLI = 0.003; Cheung and Rensvold, 2002; Meade et al., 2008).

### Criterion-Related Validity

Bivariate correlation analysis was conducted to test the relationship of variables and the criterion validity, of the general and specific factors. The results are presented in Table 7. Both the total-scale score and the three subscale scores showed good internal reliability in all samples (full sample, male, and female), with Cronbach's α ranging from 0.78[0.75, 0.81] to 0.91[0.90, 0.92]. The scores on the three subscales of the the ASDA(C) in all samples, were strongly inter-correlated (*r* ranged from 0.448 to 0.606), and had significant positive correlations (*r* ranged from 0.455 to 0.705) with the CT-DAS score. In addition, we also tested the difference in scores on the final factors between sexes. The one-way MANOVA revealed a significant multivariate main effect for sex with *F*_(3, 980)_ = 30.80, *p* < 0.001, η ^2^ = 0.086. Using the Bonferroni method for controlling Type I error rates, the follow-up univariate ANOVAs were tested at a α = 0.0167 significance level. The results indicated that female participants scored significantly higher on the three subscales than male participants did, with *F*_(1, 982)_ = 83.50, *p* < 0.001, η^2^ = 0.078 for factor “fear of dead people and tombs,” *F*_(1, 982)_ = 11.41, *p* < 0.001, η ^2^ = 0.011 for factor “fear of postmortem events,” *F*_(1, 982)_ = 28.39, *p* < 0.001, η ^2^ = 0.028 for factor “fear of lethal disease.”

**Table 7:** Correlations between factors of the ASDA and CT-DAS according to the bifactor structure.

	**Mean**	**(SD)**	**α [90%CI]**	**Subscale1**	**Subscale2**	**Subscale3**	**CT-DAS**
**Full sample(*****N*** **=** **984)**							
Total scale	46.59	13.17	0.91[.90, 0.92]	0.896^**^	0.820^**^	0.775^**^	0.661^**^
Subscale1	18.20	6.59	0.90[.89, 0.91]	–	0.606^**^	0.536^**^	0.508^**^
Subscale2	12.62	4.68	0.82[.80, 0.84]	–	–	0.479^**^	0.701^**^
Subscale3	15.77	4.43	0.80[.78, 0.82]	–	–	–	0.469^**^
**Male(*****N*** **=** **478)**							
Total scale	43.41	12.19	0.90[.89, 0.91]	0.870^**^	0.836^**^	0.755^**^	0.696^**^
Subscale1	16.31	5.98	0.90[.89, 0.91]	–	0.605^**^	0.448^**^	0.542^**^
Subscale2	12.11	4.44	0.80[.77, 0.83]	–	–	0.492^**^	0.700^**^
Subscale3	15.00	4.34	0.78[.75, 0.81]	–	–	–	0.491^**^
**Female(*****N*** **=** **506)**							
Total scale	49.59	13.37	0.91[.90, 0.92]	0.905^**^	0.811^**^	0.779^**^	0.658^**^
Subscale1	20.00	6.65	0.89[.88, 0.90]	–	0.603^**^	0.575^**^	0.509^**^
Subscale2	13.11	4.84	0.82[.80, 0.84]	–	–	0.453^**^	0.705^**^
Subscale3	16.49	4.40	0.80[.77, 0.83]	–	–	–	0.455^**^

*Subscale1, “fear of dead people and tombs”; Subscale2, “fear of postmortem events”; Subscale3, “fear of lethal disease”*.

* P < 0.05;

** *P < 0.01*.

## Summary and Discussion

Although many studies have already been carried out to investigate the structure of the ASDA, little is known about its underlying factor structure. This study was conducted in the sample of 984 Chinese college students, aimed at revealing the most optimal structure and the latent factor relations underlying the ASDA scale via bifactor modeling. After a series of comparisons, we concluded that the bifactor model provided a better fit to the sample data than the correlated traits model and the fit indices proved that within several alternative models, the bifactor model based on the newly derived 3-factor structure, was most optimal. The result also supported the measurement invariance of this bifactor structure of DA, as assessed by the ASDA, across male and female groups. At the same time, the structural analysis based on the optimal bifactor model, brought a new perspective to conceptualize the structure of DA from both general and specific components.

Some scholars maintain that death anxiety should not be a unitary or monolithic variable, on the contrary, it is complex and contains a number of components, such as fear of going to hell, loneliness and loss of identity (Karasu, 1985; Feifel, 1990). Indeed, in previous studies, it yielded different components when these unidimensional scales were subjected to factor analysis (see, e.g., Lonetto and Templer, 1986; Levin, 1990; Abdel-Khalek et al., 1993). In the current study, a series of CFA analyses were performed on the existing structure and the single-factor structure of the ASDA. The result showed that the single-factor structure presented the worst fit and all multidimensional structures fit significantly better than the single-factor structure, while the new derived 3-factor structure fit best among all structures, which was consistent with previous studies and seemed to support that the ASDA is a potentially purely multidimensional construct. However, after bifactor CFA, the superior fit of the bifactor model F suggested that there was a dominant general factor of DA underlying all the ASDA items, explaining almost two thirds of the common variance and coexists alongside three specific dimensions.

Some statistical indices associated with the bifactor model were also examined in the present study. The PUC and ECV statistic could reflect the degree to which parameter estimates will be biased when the multidimensional constructs are forced into a unidimensional model (e.g., as in a bifactor model with a general factor). In the bifactor model of this study, the PUC statistic was 70%, below the cut-off of 0.80. While Reise et al. (2013b) also stated, “when PUC is lower than 0.80, researchers may consider ECV values >0.60 and omegaH values >0.70 as tentative benchmarks.” Considering that the ECV statistic was 63%, and ω_h_ for general factor was 0.80, we came to a conclusion that although the ASDA items may be multidimensional, it would not introduce a high degree of bias on structural coefficients when fitting them into a unidimensional structure.

In fact, although the ASDA scale was developed in the multidimensional profile, many researchers still use the total score as a general index of DA in practical application. In the present study, the examination of the reliability index based on the bifactor model, lead to confidence in using the total ASDA score as a general index of DA. Generally, when the ω_h_ for subscales is sufficiently high (e.g., >0.70) and the difference between ω and ω_h_ for subscales is small (e.g., ω_h_/ω>0.70), calculating and reporting the subscale scores were justified (Reise et al., 2013a; Gu and Wen, 2017). In the current study, the general factor and the three specific factors, demonstrated good composite reliability with ω coefficients for the total ASDA score and the three subscale scores were all above 0.80. After controlling the variances accounted for in the other, the ω_h_ estimate for the general factor was 0.80, indicating that 0.80/0.94 = 85% of the reliable variance in the ASDA scores was attributable to the general factor. That meant that if 18 items were added together as a combined score, the majority of the variance will be generated by the general factor. However, after controlling the general factor, the estimated reliabilities for the summed subscale scores dropped significantly, while the differences between ω and ω-hierarchical (ω_h_/ω) for three subscales ranged from 0.24 to 0.54. One interpretation of the significant reduction was that the distinct subscales may share phenomenological similarities that are common to the ASDA in general, thus researchers should be cautious when interpreting subscale scores.

Another notable strength and novel contribution of this study, using bifactor analysis, is that the effectiveness of items could be assessed in two aspects (i.e., the loadings on the general factor and the group factor), which is difficult in traditional factor analysis. From the bifactor CFA, most of the items presented low factor loadings on the group factor after controlling the general factor. For example, item 4 presented good loadings on the general factor with 0.63, while slight loadings were presented on the corresponding group factor “fear of dead people and tombs” with 0.25. Additionally, in the traditional CFA, the factor loading of item 4 on the factor “fear of dead people and tombs” was 0.77, indicating that it was valid in measuring the general aspect of DA, while it was not adequate for measuring the target specific aspect. For item 6, the loading on the group factor “fear of lethal disease” was acceptable with 0.46, and 0.43 in the traditional CFA, while the loading on the general factor was barely satisfactory (< 0.30), which indicated that this item lacked representation in the measurement of death anxiety. Future research could assess whether the expression of this topic was distorted. In addition, there were some items such as item 2, 3, 8, 13, 14, 10, and 19, which loaded well on both the general factor and the corresponding group factor, while it presented potential cross-loading through the evaluation of modification indices, derived from the bifactor model. This result suggested that the items of the ASDA may reflect unidimensional rather than specific content.

This is the first trial to introduce an alternative structural model for the ASDA, the bifactor model, with unique advantages in validating the ASDA. The notable strength of the current study is that it has derived the optimal structure representation through a series of rigorous comparisons and assessed the underlying factor structure of DA as measured by the ASDA via a bifactor analysis. The findings should be interpreted with some caution however, as the sample was limited and raises concerns of generalizability. Thus, additional research conducted with larger and more sundry samples of participants, are required to further validate the structure of the ASDA.

## Author Contributions

QL: data analysis and paper writing; YC: paper guidance; QT: data collection; DT: method guidance.

### Conflict of Interest Statement

The authors declare that the research was conducted in the absence of any commercial or financial relationships that could be construed as a potential conflict of interest.
